# Supplementary material for: Global Transcriptomic Analysis of Topical Sodium Alginate Protection against Peptic Damage in an In Vitro Model of Treatment-Resistant Gastroesophageal Reflux Disease
Source: Int J Mol Sci. 2024 Oct 5;25(19):10714. doi: 10.3390/ijms251910714 (PMC11605242; doi:10.3390/ijms251910714)
Supplement: Supplementary file 1 [file ijms-25-10714-s001.zip › Supporting Figure S2. Tox Analysis.docx]

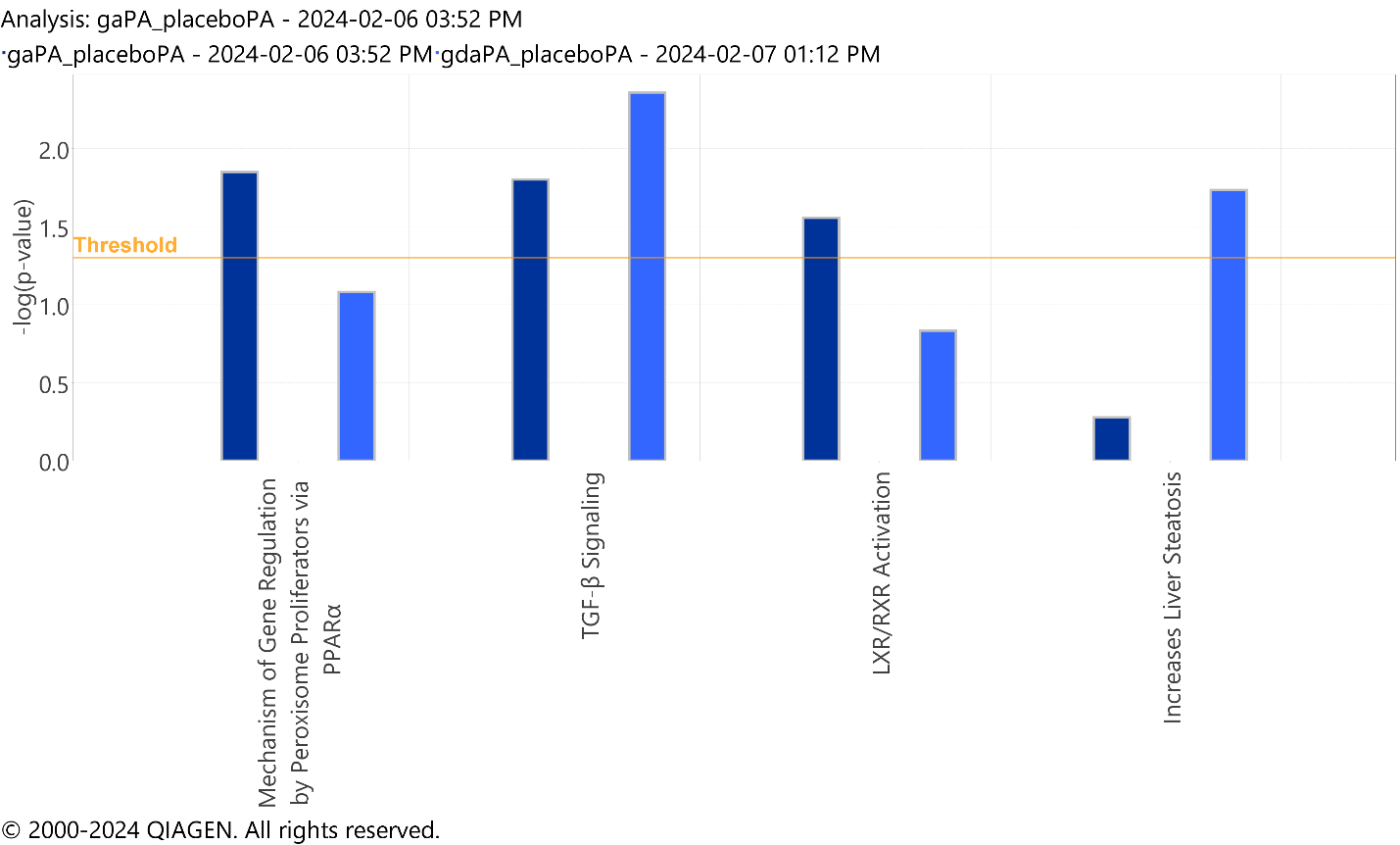


**Supporting Figure S2.** IPA Tox Function analysis. Dark blue: GA + PA vs. Placebo + PA. Blue: GDA + PA vs. Placebo + PA. Blue color indicates z-score is negative. GA = Gaviscon Advance; GDA = Gaviscon Double Action; PA = Pepsin + acid
