# Supplementary material for: Global Transcriptomic Analysis of Topical Sodium Alginate Protection against Peptic Damage in an In Vitro Model of Treatment-Resistant Gastroesophageal Reflux Disease
Source: Int J Mol Sci. 2024 Oct 5;25(19):10714. doi: 10.3390/ijms251910714 (PMC11605242; doi:10.3390/ijms251910714)
Supplement: Supplementary file 1 [file ijms-25-10714-s001.zip › Supporting Table S4. Top 10 Genes.docx]

| SUPPORTING TABLE S4.  Most Significant Differentially Expressed Transcripts of the Comparisons  Based on False Discovery Rate | | | | | | |
| --- | --- | --- | --- | --- | --- | --- |
|  | **Upregulated** | **Downregulated** |  |  | **Upregulated** | **Downregulated** |
| GA + PA vs. Placebo + PA | **Upregulated** | **Downregulated** |  | **GDA + PA vs. Placebo + PA** | MT-ND6 | FBXL15 |
|  | DNHD1 | FBXL15 |  |  | DNHD1 | JUN |
|  | LPP | MEX3D |  |  | FAT4 | MEX3D |
|  | RP11 | ATP6C |  |  | AHNAK | ATP6C |
|  | FAT1 | COL6A2 |  |  | KLF10 | COL6A2 |
|  | PTGS2 | PCSK1N |  |  | BRCA2 | PCSK1N |
|  | GPR50 | MZT2A |  |  | EXPH5 | MZT2A |
|  | CEMIP | SCAND1 |  |  | UBR4 | KLF2 |
|  | FGD6 | JUNB |  |  | PDE3A | SBNO2 |
|  | IGSF1 | TPGS1 |  |  | SPRY4 | SCAND1 |
|  |  |  |  |  |  |  |
|  | **Upregulated** | **Downregulated** |  |  |  |  |
| Placebo + PA vs. Sham + Sham | MT-ND6 | FAT4 |  |  |  |  |
|  | TPGS1 | LPP |  |  |  |  |
|  | SAPCD1 | FAT1 |  |  |  |  |
|  | KRT14 | PGAP1 |  |  |  |  |
|  | KRT13 | TRIM2 |  |  |  |  |
|  | C19orf81 | SBK3 |  |  |  |  |
|  | NICOL1 | ZBTB20 |  |  |  |  |
|  | KCNG2 | MYCBP2 |  |  |  |  |
|  | A1BG | CSMD1 |  |  |  |  |
|  | SPRR2A | DGKH |  |  |  |  |
|  |  |  |  |  |  |  |
