# Supplementary figures and images for: Global Transcriptomic Analysis of Topical Sodium Alginate Protection against Peptic Damage in an In Vitro Model of Treatment-Resistant Gastroesophageal Reflux Disease
Source: Int J Mol Sci. 2024 Oct 5;25(19):10714. doi: 10.3390/ijms251910714 (PMC11605242; doi:10.3390/ijms251910714)

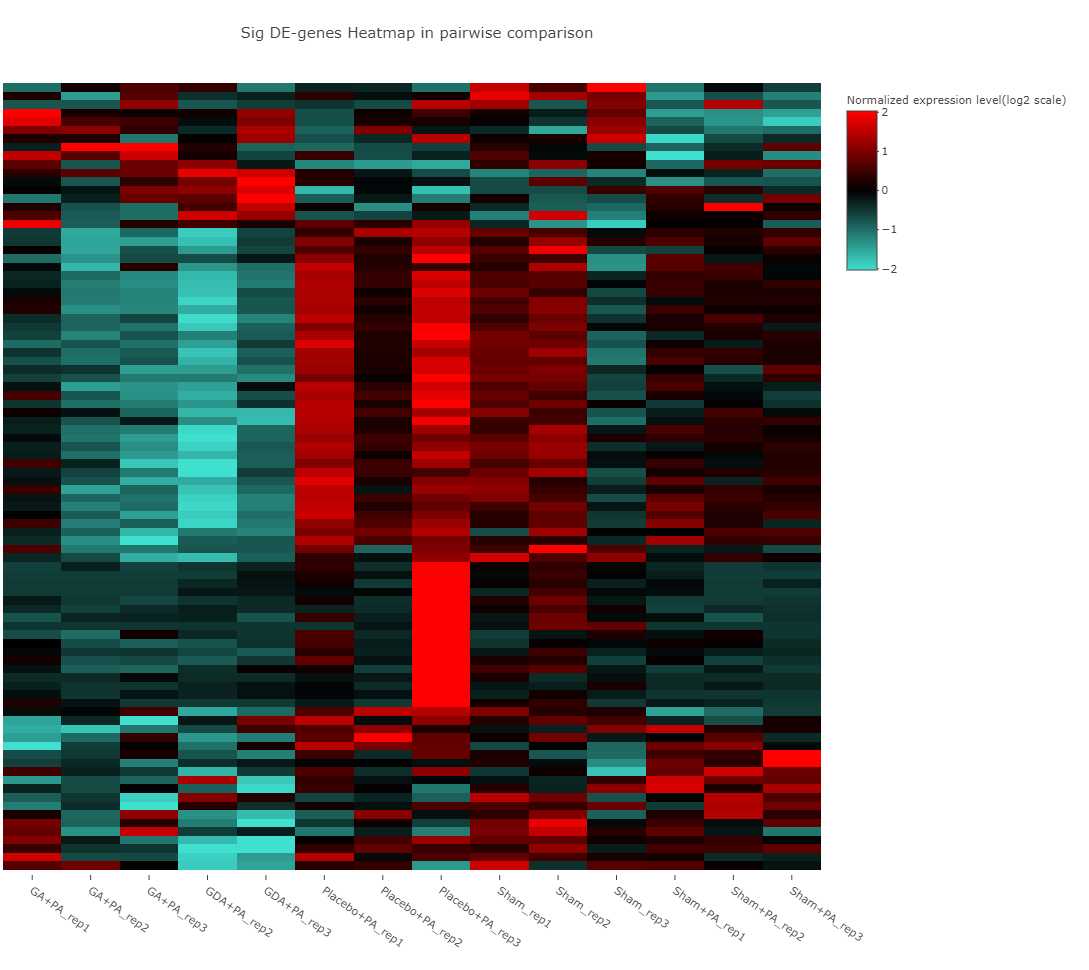

Supplement: Supplementary file 1 [file ijms-25-10714-s001.zip › Supporting Figure S1. Heat map.png]
